# Crystal structure of 1-methyl-4-methyl­sulfanyl-1*H*-pyrazolo­[3,4-*d*]pyrimidine

**DOI:** 10.1107/S1600536814025239

**Published:** 2014-11-21

**Authors:** Mohammed El Fal, Youssef Ramli, El Mokhtar Essassi, Mohamed Saadi, Lahcen El Ammari

**Affiliations:** a Laboratoire de Chimie Organique Hétérocyclique URAC 21, Pôle de Compétences Pharmacochimie, Av. Ibn Battouta, BP 1014, Faculté des Sciences, Université Mohammed V, Rabat, Morocco; b Medicinal Chemistry Laboratory, Faculty of Medicine and Pharmacy, Mohammed V University, Rabat, Morocco; c Laboratoire de Chimie du Solide Appliquée, Faculté des Sciences, Université Mohammed V, Avenue Ibn Battouta, BP 1014, Rabat, Morocco

**Keywords:** crystal structure, 1*H*-pyrazolo­[3,4-*d*]pyrimidine, pharmacol­ogical and biochemical properties, π–π inter­actions

## Abstract

In the title compound, C_7_H_8_N_4_S, the non-H atoms of the pyrazolo­[3,4-*d*]pyrimidine ring system and the methyl­sulfanyl group lie on a crystallographic mirror plane. In the crystal, mol­ecules are linked *via* a number of π–π inter­actions [centroid–centroid distances vary from 3.452 (7) to 3.6062 (8) Å], forming a three-dimensional structure.

## Related literature   

For similar compounds, see: El Fal *et al.* (2013 ▶, 2014*a* ▶,*b* ▶); Ouzidan *et al.* (2011 ▶). For pharmacological and biochemical properties of pyrazolo­[3,4-*d*]pyrimidine-4(5*H*)-thione derivatives, see: Chauhan & Kumar (2013 ▶); Venkatesan *et al.* (2014 ▶); Rashad *et al.* (2011 ▶).
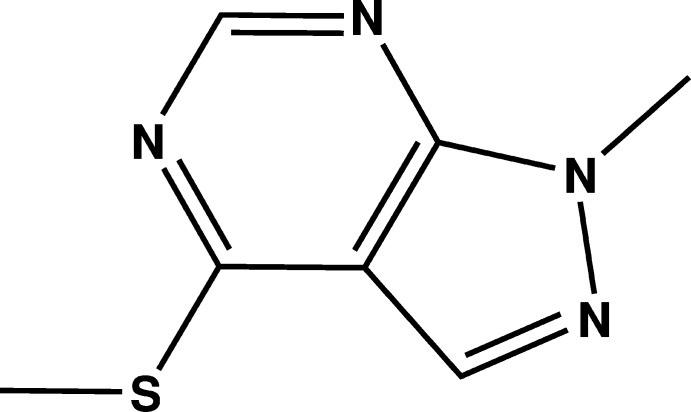


## Experimental   

### Crystal data  

C_7_H_8_N_4_S
*M* *_r_* = 180.23
Orthorhombic, 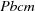

*a* = 7.9309 (14) Å
*b* = 15.335 (3) Å
*c* = 6.7158 (12) Å
*V* = 816.8 (3) Å^3^
*Z* = 4
Mo *K*α radiation
μ = 0.34 mm^−1^
*T* = 296 K
0.37 × 0.28 × 0.19 mm

### Data collection  

Bruker X8 APEX diffractometer
Absorption correction: multi-scan (*SADABS*; Bruker, 2009 ▶) *T*_min_ = 0.637, *T*_max_ = 0.746
2970 measured reflections
1227 independent reflections
1017 reflections with *I* > 2σ(*I*)
*R*_int_ = 0.017

### Refinement  

*R*[*F* ^2^ > 2σ(*F* ^2^)] = 0.042
*wR*(*F* ^2^) = 0.129
*S* = 1.09
1227 reflections
73 parameters
H-atom parameters constrained
Δρ_max_ = 0.44 e Å^−3^
Δρ_min_ = −0.29 e Å^−3^

### 

Data collection: *APEX2* (Bruker, 2009 ▶); cell refinement: *SAINT* (Bruker, 2009 ▶); data reduction: *SAINT*; program(s) used to solve structure: *SHELXS97* (Sheldrick, 2008 ▶); program(s) used to refine structure: *SHELXL97* (Sheldrick, 2008 ▶); molecular graphics: *ORTEP-3 for Windows* (Farrugia, 2012 ▶); software used to prepare material for publication: *PLATON* (Spek, 2009 ▶) and *publCIF* (Westrip, 2010 ▶).

## Supplementary Material

Crystal structure: contains datablock(s) I. DOI: 10.1107/S1600536814025239/tk5348sup1.cif

Structure factors: contains datablock(s) I. DOI: 10.1107/S1600536814025239/tk5348Isup2.hkl

Supporting information file. DOI: 10.1107/S1600536814025239/tk5348Isup3.cml

**Fig. 1.**
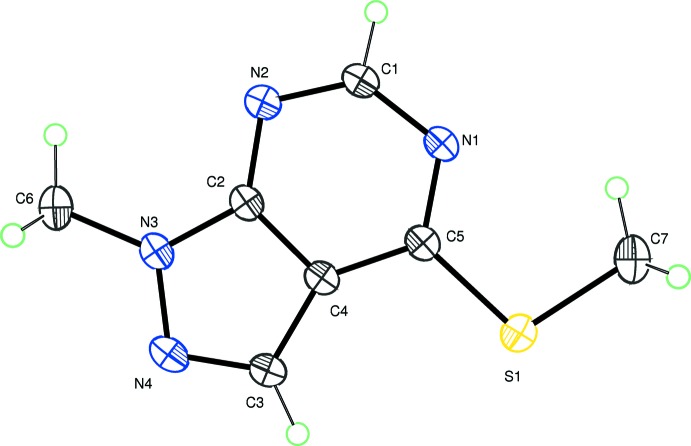
Molecular structure of the title compound with the atom-labelling scheme. Displacement ellipsoids are drawn at the 50% probability level. H atoms are represented as small circles.

. DOI: 10.1107/S1600536814025239/tk5348fig1.tif
Mol­ecular structure of the title compound with the atom-labelling scheme. Displacement ellipsoids are drawn at the 50% probability level. H atoms are represented as small circles.

CCDC reference: 1034638

Additional supporting information:  crystallographic information; 3D view; checkCIF report

## supplementary crystallographic information

### S1. Structural commentary

Synthesis of 1*H*-pyrazolo [3,4-*d*] pyrimidine-4-thiol derivatives has received considerable attention due to their biological activity especially as anti­microbial (Chauhan *et al.*, 2013), anti­tubercular (Venkatesan *et al.*, 2014) and anti­cancer (Rashad *et al.*, 2011) agents. During the search for new anti­bacterial agents synthesis, some 1*H*-pyrazolo­[3,4-*d*] pyrimidine-4-thiol derivatives were prepared (El Fal *et al.*, 2013, 2014*a*, 2014*b*; Ouzidan *et al.*, 2011). These compounds are currently under investigation for possible biological activity.

The molecule of the title compound is build up from two fused five- and six-membered rings linked to methyl­sulfanyl group. All non hydrogen atoms of the molecule are coplanar as shown in Fig. 1. In the crystal, the molecules are linked together by a number of π–π inter­actions [centroid–centroid distances vary from 3.452 (7) to 3.6062 (8) Å], forming a three-dimensional structure.

### S2. Synthesis and crystallization

To a solution of 1*H*-pyrazolo [3,4-*d*] pyrimidine-4-thiol (0.5 g, 3.28 mmol) dissolved in DMF (20 ml) was added iodo­methane (0.43 ml, 6.62 mmol), potassium carbonate (0.93 g, 7.1 mmol) and a catalytic amount of tetra-n-butyl­ammonium bromide (0.1 g, 0.4 mmol). The mixture was stirred for 48 h and monitored by thin layer chromatography. The mixture was filtered and the solvent was removed *in vacuo*. The solid obtained was crystallized from ethanol to give the title compound as orange crystals (yield: 65%).

### S3. Refinement

The H atoms were located in a difference map and treated as riding with C—H = 0.93 Å (aromatic) and C—H = 0.96 Å (methyl), and with *U*_iso_(H) = 1.2 *U*_eq_ (aromatic) and *U*_iso_(H) = 1.5 *U*_eq_ (methyl).

### Crystal data

**Table:**

C_7_H_8_N_4_S	*F*(000) = 376
*M**_r_* = 180.23	*D*_x_ = 1.466 Mg m^−^^3^
Orthorhombic, *P**b**c**m*	Mo *K*α radiation, λ = 0.71073 Å
Hall symbol: -P 2c 2b	Cell parameters from 1227 reflections
*a* = 7.9309 (14) Å	θ = 2.6–29.6°
*b* = 15.335 (3) Å	µ = 0.34 mm^−^^1^
*c* = 6.7158 (12) Å	*T* = 296 K
*V* = 816.8 (3) Å^3^	Block, orange
*Z* = 4	0.37 × 0.28 × 0.19 mm

### Data collection

**Table:**

Bruker X8 APEX diffractometer	1227 independent reflections
Radiation source: fine-focus sealed tube	1017 reflections with *I* > 2σ(*I*)
Graphite monochromator	*R*_int_ = 0.017
φ and ω scans	θ_max_ = 29.6°, θ_min_ = 2.6°
Absorption correction: multi-scan (*SADABS*; Bruker, 2009)	*h* = −10→4
*T*_min_ = 0.637, *T*_max_ = 0.746	*k* = −21→19
2970 measured reflections	*l* = −3→9

### Refinement

**Table:**

Refinement on *F*^2^	Primary atom site location: structure-invariant direct methods
Least-squares matrix: full	Secondary atom site location: difference Fourier map
*R*[*F*^2^ > 2σ(*F*^2^)] = 0.042	Hydrogen site location: inferred from neighbouring sites
*wR*(*F*^2^) = 0.129	H-atom parameters constrained
*S* = 1.09	*w* = 1/[σ^2^(*F*_o_^2^) + (0.0694*P*)^2^ + 0.3181*P*] where *P* = (*F*_o_^2^ + 2*F*_c_^2^)/3
1227 reflections	(Δ/σ)_max_ < 0.001
73 parameters	Δρ_max_ = 0.44 e Å^−^^3^
0 restraints	Δρ_min_ = −0.29 e Å^−^^3^

### Special details

**Table:**

Geometry. All e.s.d.'s (except the e.s.d. in the dihedral angle between two l.s. planes) are estimated using the full covariance matrix. The cell e.s.d.'s are taken into account individually in the estimation of e.s.d.'s in distances, angles and torsion angles; correlations between e.s.d.'s in cell parameters are only used when they are defined by crystal symmetry. An approximate (isotropic) treatment of cell e.s.d.'s is used for estimating e.s.d.'s involving l.s. planes.
Refinement. Refinement of *F*^2^ against all reflections. The weighted *R*-factor *wR* and goodness of fit *S* are based on *F*^2^, conventional *R*-factors *R* are based on *F*, with *F* set to zero for negative *F*^2^. The threshold expression of *F*^2^ > σ(*F*^2^) is used only for calculating *R*-factors(gt) *etc*. and is not relevant to the choice of reflections for refinement. *R*-factors based on *F*^2^ are statistically about twice as large as those based on *F*, and *R*- factors based on all data will be even larger.

### Fractional atomic coordinates and isotropic or equivalent isotropic displacement parameters (Å^2^)

**Table:**

	*x*	*y*	*z*	*U*_iso_*/*U*_eq_	
C1	0.5519 (3)	0.06804 (13)	0.2500	0.0393 (5)	
H1	0.4928	0.0156	0.2500	0.047*	
C2	0.5516 (3)	0.21199 (12)	0.2500	0.0279 (4)	
C3	0.7675 (3)	0.30515 (13)	0.2500	0.0322 (4)	
H3	0.8763	0.3277	0.2500	0.039*	
C4	0.7284 (2)	0.21512 (11)	0.2500	0.0264 (4)	
C5	0.8115 (3)	0.13396 (11)	0.2500	0.0285 (4)	
C6	0.3247 (3)	0.32714 (15)	0.2500	0.0450 (6)	
H6A	0.3121	0.3658	0.1386	0.068*	
H6B	0.2414	0.2818	0.2500	0.068*	
C7	1.0858 (3)	0.02097 (16)	0.2500	0.0483 (6)	
H7A	0.9865	−0.0148	0.2500	0.072*	
H7B	1.1499	0.0095	0.1316	0.072*	
N1	0.7221 (2)	0.06053 (10)	0.2500	0.0348 (4)	
N2	0.4577 (2)	0.13910 (11)	0.2500	0.0369 (4)	
N3	0.4970 (2)	0.29563 (11)	0.2500	0.0323 (4)	
N4	0.6289 (3)	0.35277 (10)	0.2500	0.0349 (4)	
S1	1.03110 (7)	0.13414 (4)	0.2500	0.0447 (2)	

### Atomic displacement parameters (Å^2^)

**Table:**

	*U*^11^	*U*^22^	*U*^33^	*U*^12^	*U*^13^	*U*^23^
C1	0.0316 (10)	0.0218 (9)	0.0644 (15)	−0.0024 (7)	0.000	0.000
C2	0.0294 (9)	0.0221 (8)	0.0321 (9)	0.0016 (7)	0.000	0.000
C3	0.0301 (10)	0.0230 (8)	0.0434 (11)	−0.0032 (7)	0.000	0.000
C4	0.0280 (9)	0.0216 (8)	0.0295 (9)	−0.0008 (6)	0.000	0.000
C5	0.0295 (9)	0.0232 (9)	0.0329 (9)	0.0005 (7)	0.000	0.000
C6	0.0329 (11)	0.0330 (11)	0.0692 (16)	0.0095 (9)	0.000	0.000
C7	0.0376 (12)	0.0393 (12)	0.0680 (17)	0.0119 (10)	0.000	0.000
N1	0.0307 (9)	0.0199 (7)	0.0536 (11)	0.0005 (6)	0.000	0.000
N2	0.0290 (9)	0.0250 (9)	0.0568 (12)	−0.0015 (6)	0.000	0.000
N3	0.0318 (8)	0.0221 (7)	0.0429 (9)	0.0024 (6)	0.000	0.000
N4	0.0399 (10)	0.0211 (7)	0.0439 (10)	−0.0021 (6)	0.000	0.000
S1	0.0264 (3)	0.0316 (3)	0.0761 (5)	0.00110 (18)	0.000	0.000

### Geometric parameters (Å, º)

**Table:**

C1—N2	1.321 (3)	C5—N1	1.331 (2)
C1—N1	1.355 (3)	C5—S1	1.741 (2)
C1—H1	0.9300	C6—N3	1.449 (3)
C2—N2	1.343 (3)	C6—H6A	0.9598
C2—N3	1.354 (2)	C6—H6B	0.9599
C2—C4	1.403 (3)	C7—S1	1.789 (3)
C3—N4	1.319 (3)	C7—H7A	0.9600
C3—C4	1.415 (2)	C7—H7B	0.9599
C3—H3	0.9300	N3—N4	1.365 (3)
C4—C5	1.408 (2)		
			
N2—C1—N1	129.3 (2)	C4—C5—S1	117.82 (14)
N2—C1—H1	115.3	N3—C6—H6A	107.7
N1—C1—H1	115.3	N3—C6—H6B	114.1
N2—C2—N3	127.68 (18)	H6A—C6—H6B	112.1
N2—C2—C4	125.63 (18)	S1—C7—H7A	110.8
N3—C2—C4	106.69 (17)	S1—C7—H7B	107.8
N4—C3—C4	110.97 (17)	H7A—C7—H7B	109.3
N4—C3—H3	124.5	C5—N1—C1	117.33 (17)
C4—C3—H3	124.5	C1—N2—C2	111.89 (18)
C2—C4—C5	115.96 (17)	C2—N3—N4	111.29 (17)
C2—C4—C3	104.60 (16)	C2—N3—C6	128.13 (19)
C5—C4—C3	139.45 (19)	N4—N3—C6	120.59 (17)
N1—C5—C4	119.88 (19)	C3—N4—N3	106.45 (16)
N1—C5—S1	122.30 (14)	C5—S1—C7	103.95 (11)
