# Supplementary figures and images for: Crystal structure of 1-methyl-4-methyl­sulfanyl-1H-pyrazolo­[3,4-d]pyrimidine
Source: Acta Crystallogr Sect E Struct Rep Online. 2014 Nov 21;70(Pt 12):o1281. doi: 10.1107/S1600536814025239 (PMC4257437; doi:10.1107/S1600536814025239)

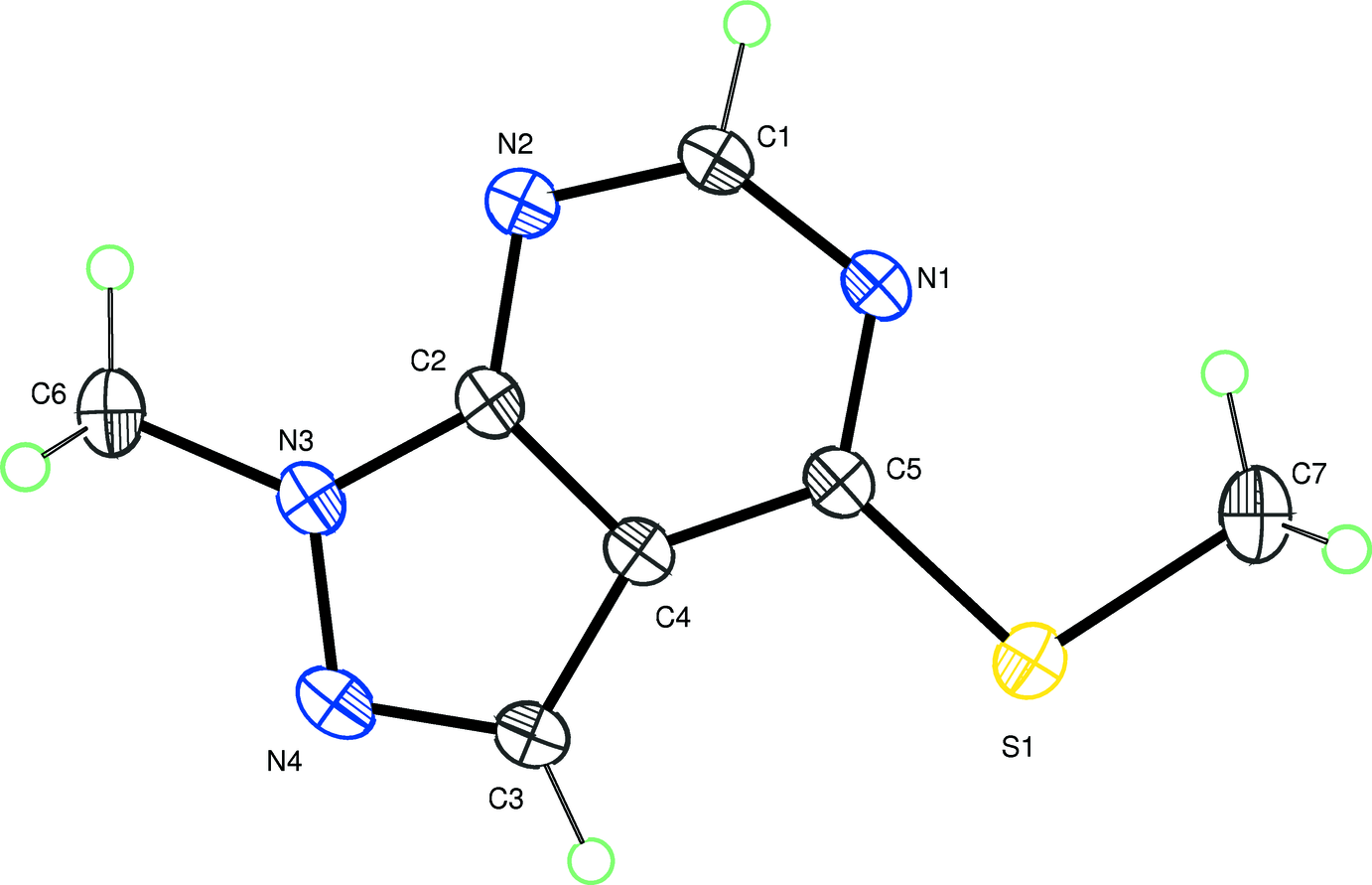

Supplement: Supplementary file 4 [file e-70-o1281-fig1.tif]
